# An Ongoing Process: The Implementation of an Intervention for People With Profound Intellectual and Multiple Disabilities Over Time

**DOI:** 10.1111/jar.70098

**Published:** 2025-07-16

**Authors:** Josien Schaafsma, Annet ten Brug, Annette van der Putten

**Affiliations:** ^1^ Faculty of Behavioural and Social Sciences, Department of Pedagogy and Educational Sciences, Basic Unit of Inclusive and Special Needs Education University of Groningen Groningen the Netherlands

## Abstract

**Introduction:**

The “Programma Perspectief” intervention aims to provide optimal support to people with profound intellectual and multiple disabilities. Knowledge concerning intervention fidelity is outdated and scarce as is typical for the support of persons with special needs.

**Methods:**

An survey amongst care professionals (*n* = 62) yielded data on the application of core elements of the intervention (vision, methodical approach, and collaboration) in practise, as well as on implementation barriers and facilitators.

**Results:**

Vision and methodical approach are reflected in practise. A positive association was found between the number of perceived barriers and time since implementation. Over time, more barriers emerged at organisational level.

**Conclusions:**

The results reveal the importance of attending to implementation, even after years. Further research should focus on developing focused implementation strategies to enhance the sustainability of the analysed intervention, thereby guaranteeing the quality of support provided to people with profound intellectual and multiple disabilities.

**Summary**

- The results indicate that time since implementation does not relate to the extent in which professionals applied the core elements of the intervention Programma Perspectief.
- Interdisciplinary collaboration is vulnerable: the study shows that professionals generally collaborate with colleagues within their own disciplines, but to a lesser extent with colleagues from other disciplines and with relatives.
- Time since implementation related to factors influencing intervention fidelity. Different types of barriers and facilitators were perceived as time passes: more barriers were perceived in organisations where implementation had taken place less recently.
- This study demonstrates that the implementation of an intervention is an ongoing process. The decision to sustain its use should be a deliberate and strategic choice, warranting continuous organisational attention, as new challenges will inevitably arise over time.

## 1 Introduction

Because of their intensive, specialised, and lifelong support needs, the quality of life of people with profound intellectual and multiple disabilities is highly dependent on support provided by others (Buntinx and Schalock 2010; Niedbalski 2023; Petry et al. 2007; Schalock et al. 2021; Van der Putten et al. 2017). People with profound intellectual and multiple disabilities have a combination of a profound intellectual disability and a severe or profound motor disability, often combined with additional sensory and health impairments (Van Timmeren et al. 2017). Their communication is often non‐verbal and subtle, and it can differ according to context (Nakken and Vlaskamp 2007). Given these support needs and ways of communicating, the provision of support adapted to a person's needs and abilities is a challenge (Van der Putten et al. 2017; Vlaskamp et al. 2005). According to a recent study, care professionals experience a range of such challenges in several areas. For example, with regard to interacting with the person, professionals are likely to have difficulty interpreting the communicative behaviour of the person and identifying their specific individual needs. With regard to their own functioning, professionals experience that they do not have enough knowledge to provide sufficient support to these people. At the organisational level, challenges encountered by professionals include lack of time and difficulty collaborating with others (De Jong et al. 2022).

To provide appropriate support for people with profound intellectual and multiple disabilities and to meet these perceived challenges, an Individualised support program or educational program known as “Programma Perspectief” has been developed in recent decades (Vlaskamp 1993; Vlaskamp et al. 2015, 2005; Van der Putten et al. 2009; Vlaskamp and van der Putten 2009). This intervention consists of several interconnected core elements addressing the challenges related to the support of persons with multiple disabilities. The first element is a normative statement (vision) that people with profound intellectual and multiple disabilities are capable of engaging in and maintaining meaningful relationships with others. Within these relationships, they can make their needs and desires known and develop and exert influence over their existence (Vlaskamp et al. 2005). The normative statement is transferred into daily practise by working in a methodical, goal‐oriented manner (the second core element). The intervention requires interdisciplinary collaboration between professionals, as well as between professionals and relatives (the third core element). Relatives often play an important role in the lives of people with profound intellectual and multiple disabilities, and they therefore possess a large volume of person‐specific knowledge (Kruithof et al. 2020).

The methodical approach consists of a working model composed of several consecutive steps in order to translate the rather general vision and interdisciplinarity into practise. First, a personal profile is set up based on information obtained from all people involved in providing support to the person. This profile is used to formulate interdisciplinary long‐term goals (one for 1–2 years reached by one or more for 6–12 months) and monodisciplinary short‐term goals (4–6 weeks). These goals are formulated and evaluated after a fixed period (Van der Putten et al. 2009). The intervention is cyclical in nature; the evaluation of the goals is followed by a new developmental perspective. A comprehensive implementation plan is available, including strategies and materials that help optimise implementation (Vlaskamp et al. 2005, 2015; Vlaskamp and van der Putten 2009; Zijlstra et al. 2005).

The intervention is one of the few evidence‐based interventions available for people with profound intellectual and multiple disabilities (Maes et al. 2021; Vlaskamp et al. 2015). Studies have indicated that the intervention has effects for people with profound intellectual and multiple disabilities (e.g., perceived improvement in communication and mood), care professionals (e.g., increased knowledge, skills and working in a methodical and goal‐oriented way), organisations (e.g., increased collaboration within and between professional disciplines), and relatives (e.g., enhanced understanding of the relative with disabilities and greater insight into the support provided) (Bakker and Munde 2007; Luijkx and Metsemakers 2008; Pruis 2010; Poppes and Vlaskamp 2001; Vlaskamp and van der Putten 2009; Vlaskamp et al. 2015). Over the years, several studies have been conducted on the implementation of the intervention, and various barriers to and facilitators of implementation have been distinguished with regard to the intervention, care professionals, the organisation, people with profound intellectual and multiple disabilities, and the implementation process (Bakker and Munde 2007; Luijkx and Metsemakers 2008; Vlaskamp et al. 2005; Zijlstra 2003).

These earlier studies contribute to the small body of evidence that now exists about implementation processes and strategies in the support for people with profound intellectual and multiple disabilities. Despite the fact that there is research on the effectiveness of Programma Perspectief (Bakker and Munde 2007; Luijkx and Metsemakers 2008; Pruis 2010; Poppes and Vlaskamp 2001; Vlaskamp and van der Putten 2009; Vlaskamp et al. 2015), and the intervention has proven to produce a desired result outside an artificial controlled setting, the existing body of evidence also has several shortcomings. The research conducted on the implementation of this intervention is relatively outdated, and it focuses primarily on the initial implementation (short‐term, i.e., within 2 years) and, to a lesser extent, on fidelity over time (Vlaskamp et al. 2015). By fidelity we mean the degree in which the core elements (or key components) of an intervention are applied in practise as theoretically intended (An et al. 2020). The full implementation of evidence‐based interventions is commonly estimated to take 2–4 years. After that period, the focus shifts to sustaining the intervention (Fixsen 2005). To ensure the continuity of an intervention and its intended effects in the long term, it is important also to focus on the use of the intervention over time (Shelton et al. 2018). It is known that many evidence‐based interventions that have been implemented are not sustained over time, and intervention drift (unintentionally deviating from the theoretically defined core components) may occur (Birken et al. 2020; Boswell and Schwartzman 2024; Glasgow et al. 2003; Hailemariam et al. 2019). Using a dynamic sustainability framework, we acknowledge that changes occur in interventions over time influenced by the practise setting and broader ecological setting (Chambers et al. 2013). Scholars are increasingly recognising that the sustainability of executing the intervention as intended is influenced by several factors relating to the intervention (e.g., the absence of attractive alternatives), professionals (e.g., training), and the organisation (e.g., organisational culture, peer‐to‐peer feedback opportunities, and adequate financial resources) (Daamen 2015; Doyle et al. 2013; Shelton et al. 2018; Wiltsey Stirman et al. 2012; Zurynski et al. 2023). In light of these observations, it is important to gain insight into the sustainability of the intervention, especially after the initial implementation period. In doing so, it is relevant to analyse what changes have taken place over time and whether core elements are still used as theoretically described or are lost or adjusted over time.

The current study aims to provide insight into the intervention fidelity, the application of core elements of Programma Perspectief in current practise. This contributes to the body of evidence surrounding existing implementation science literature on complex multifaceted interventions, specifically within the support of people with profound intellectual and multiple disabilities. We analysed to what extent the core elements are still used in practise and explored the relationship between the application of these core elements and time since implementation. This knowledge could identify possible areas for improvement in implementation to ensure long‐term effectiveness. Results also contribute to the body of knowledge concerning interventions and implementation processes in the support of persons with intellectual disabilities.

The following research questions have been formulated:

1. To what extent are the core elements of the intervention applied in practise?
2. What is the association between the application of the core elements and the perceived effects for the person with profound intellectual and multiple disabilities?
3. What is the association between time since implementation and the application of the core elements, the perceived barriers and facilitators, and the perceived effects?

## 2 Materials and Methods

### 2.1 Context and Design

The research was conducted by the Academic Collaborative Centre for People with Profound Intellectual and Multiple Disabilities (ACC PIMD); a collaboration between the University of Groningen, the Hanze University of Applied Sciences Groningen, and organisations that provide support to people with profound intellectual and multiple disabilities. A cross‐sectional study was conducted using a structured online questionnaire (Hennink et al. 2011; Wouters and Aarts 2016).

### 2.2 Respondents

The target group consists of care professionals working as direct support professionals, health care psychologists^1^, allied healthcare professionals, managers, team leaders, physicians, or nurses. The inclusion criteria were as follows:

- Employed in a healthcare organisation in the Netherlands that provides support to people with profound intellectual and multiple disabilities (Nakken and Vlaskamp 2007)^2^ and that has implemented Programma Perspectief
- Involved in providing support to people with profound intellectual and multiple disabilities
- Working with Programma Perspectief, meaning that the professional works according to at least one of the core elements (e.g., setting goals, collaborating with colleagues from other disciplines and with relatives, or working according to the normative statement).

### 2.3 Procedure

The Ethics Committee Behavioural and Social Sciences of the University of Groningen granted approval to conduct the study on 8 October 2023 (UG‐2223‐GMW‐S‐000007). Data collection took place from November 2023 to February 2024.

The online questionnaire was administered to healthcare organisations within the network of the ACC PIMD. The healthcare organisations participating in this study sent the questionnaire to their employees. In addition, the questionnaire was also distributed through various channels of the ACC PIMD, including email, website, and social media. Information letters were sent along with information on the purpose of the study, intended target group, voluntary participation, data management, and dissemination of the results. Informed written consent was obtained from all respondents prior to participation in the questionnaire. During data collection, healthcare organisations were asked to send a reminder, thereby once more inviting their employees to participate.

### 2.4 Data Collection and Instruments

Insight into the application of Programma Perspectief in current practise was generated with a questionnaire based on the existing literature and in close consultation with relevant stakeholders such as professionals. First, when the research proposal was written, a project group containing health care psychologists, direct support professionals, allied healthcare professionals, a team lead, a manager, and a trainer was formed and gave feedback on the proposal containing the outline of the questionnaire. During the study, a draft questionnaire was created, to which the project group provided meticulous written feedback. The questionnaire was then discussed during a 2‐h focus group with the project group. Finally, the penultimate version was completed by two participants whilst the researcher was there too. During the completion process, the participants always thought aloud. Based on this session, the researcher removed ambiguities from the concept version of the questionnaire.

The questionnaire consists of five parts. The first part (16 items) assesses whether respondents meet the inclusion criteria of the study and collects information on the background characteristics of the professionals, the people they support, and the organisation in which they work. These variables were based on earlier studies into the effectiveness and implementation of the intervention (Zijlstra 2003; Vlaskamp and van der Putten 2009) and the description of the intervention (Vlaskamp et al. 2015, 2006).

The second part of the questionnaire consists of 18 items related to characteristics of the implementation, and it is based on strategies for the optimal implementation of Programma Perspectief found in earlier studies and based on (Vlaskamp and van der Putten 2009; Vlaskamp et al. 2015; Zijlstra et al. 2005). The items address when and how the implementation took place (e.g., “When you started working with Programma Perspectief, did you attend a training or a course”?). The items consist of open‐ended questions with numerical response options (years) and multiple‐choice questions.

The third part of the questionnaire is composed of 27 items relating to the application of the core elements and the extent to which respondents apply those elements in practise. The following is an example of an item: “I collaborate with other colleagues outside of my own discipline”. The items consist of statements with four‐point Likert scales (e.g., never/sometimes/usually/always), two‐point Likert scales (yes/no), multiple‐choice questions, and open‐ended questions.

The fourth part of the questionnaire consists of 38 items on barriers and facilitators related to working with the intervention, based on the evidence‐based Measurement Instrument for Determinants of Innovations (MIDI) (Fleuren et al. 2012, 2014). In accordance with the MIDI guidelines, several adaptations were made to make the MIDI fit the topic and context of the current study. Some items were omitted because they were either not applicable (e.g., the item “Clients will generally be satisfied if I use this innovation”) or not relevant (e.g., items relating to the socio‐political environment). Several items were added based on existing literature on implementation factors and Programma Perspectief, including “There is enough interdisciplinary collaboration to work with Programma Perspectief as intended” (Vlaskamp et al. 2005; Zijlstra 2003; Zijlstra et al. 2005). All items are based on Likert scales with two (yes/no) to five (e.g., completely disagree to completely agree) response options. The fourth part of the questionnaire concludes with two open‐ended questions in which respondents can describe additional barriers and facilitators.

The fifth and final part of the questionnaire is composed of 28 items on the perceived effects of the intervention for the person with profound intellectual and multiple disabilities, the professional, and the organisation, based on the *Vragenlijst Evaluatie Werkwijze* (Methodology Evaluation Questionnaire) (Vlaskamp 1993; Vlaskamp et al. 2006). The items are based on Likert scales with three or four response options (effect perceived in majority/minority/none of the people; completely disagree to completely agree).

Not all respondents were presented with the same items, as some items differed by professional role or were presented based on previous responses. The digital final version of the questionnaire was tested by two professionals, who provided feedback on functionality, clarity, vocabulary, and length. Examples of adjustments made as a result of this feedback include changing the item “I have interdisciplinary consultations about formulating and evaluating a client's long‐term goal for 1–2 years” to “I have consultations with colleagues from other disciplines and with relatives about formulating and evaluating a client's long‐term goal for 1–2 years” and adding the response option “don't know (anymore)” to a number of items.

### 2.5 Analysis

Data were prepared and analysed using the Statistical Package for the Social Sciences (SPSS) version 28. Only respondents who had completed the questionnaire from start to finish were included in the analysis.

Prior to analysis, the dataset was prepared. For the items with Likert scales, reverse coding was applied to negative items such that higher scores represent a higher degree of applying the core elements, a more positive contribution to implementation, or more perceived effects. For MIDI items measuring barriers and facilitators, positive responses were merged (e.g., “completely agree” and “agree”), as were negative responses. In addition, several new variables were created. For time since implementation, a dummy variable was created with the categories “less than two years ago” (more recently) and “two or more years ago” (less recently). The choice for the split at 2 years is based on existing knowledge on the implementation of the intervention and the sustainable implementation of evidence‐based interventions in general (Fixsen 2005; Vlaskamp et al. 2015). In addition, two ratio variables were created, based on all closed‐ended MIDI items measuring barriers and facilitators: the total number of perceived barriers for each respondent, and the total number of facilitators for each respondent. To calculate the total number for each respondent, a MIDI item was perceived as a barrier if the respondent disagreed (or completely disagreed) and as a facilitator if the respondent agreed (or completely agreed). Three additional ratio variables were created for the total number of perceived effects for the person with profound intellectual and multiple disabilities, the professional, and the organisation, respectively. See Supporting Information 1 for the underlying items and the calculation of the scores for these three variables.

Descriptive statistics were used to describe the extent to which the core elements of the intervention were applied in practise (Research Question 1). Inductive analysis was applied to all open‐ended items, with a response (or category) being included if it was mentioned by respondents at least twice. To analyse whether the application of core elements relates to the number of perceived effects for the person with profound intellectual and multiple disabilities (Research Question 2), bivariate statistics were reported. Furthermore, descriptive and bivariate statistics were presented to indicate differences based on time since implementation in the application of the core elements, the type and number of perceived barriers to and facilitators of implementation, and the number of perceived effects for the person with profound intellectual and multiple disabilities, the professional, and the organisation (Research Question 3). A MIDI item was identified as a facilitator if ≥ 80% of the respondents agreed (or completely agreed) with the item, and as a barrier if ≥ 20% disagreed (or completely disagreed) (Van Dam et al. 2022; Deenik et al. 2019; Schepers et al. 2017; Verberne et al. 2018). Non‐parametric tests (Chi‐square tests, Mann–Whitney *U* tests, and Spearman correlations) were applied to the bivariate statistics, given the sample size and the non‐normal distribution of variables, with *p*‐values < 0.05 considered statistically significant. To enhance readability, only significant results on the bivariate analyses are reported in the Results section. An overview of all significant and non‐significant results is included in Supporting Information 3.

## 3 Results

The questionnaire was completed by 62 respondents. Initially, a total of 100 respondents opened the questionnaire and gave consent to participate. Of these, 11 did not work with Programma Perspectief and therefore did not meet the inclusion criteria. Of the remaining 89 respondents, a total of 27 respondents answered only a few questions (e.g., related to their job) and then stopped the questionnaire. Characteristics of the 62 respondents, the age group of the people with profound intellectual and multiple disabilities to which they provide support, and the type and size of the organisations in which they work are presented in Table 1.

**TABLE 1:** Characteristics of respondents, people with profound intellectual and multiple disabilities, and organisations.

Characteristics	*n* (%)	Mean [SD], min–max
Characteristics of respondents (*n* = 62)		
(Primary) profession/role		
Direct support professional	25 (40.3)	
Health care psychologist	20 (32.3)	
Manager or team lead	7 (11.3)	
Allied healthcare professionals	5 (8.1)	
Physician	2 (3.2)	
Nurse	2 (3.2)	
Trainer	1 (1.6)	
Level of education		
Intermediate vocational education	27 (43.5)	
Higher vocational education	12 (19.4)	
Higher education	23 (37.1)	
Age (in years)		43 [12], 21–64
Duration of employment in the organisation (in years)		12 [10], 1–35
Characteristics of people with profound intellectual and multiple disabilities (*n* = 55)^a^		
Age group (in years)^b^		
< 18	26 (47.3)	
18–49	46 (83.6)	
≥ 50	42 (76.4)	
Characteristics of the organisation (*n* = 62)		
Type of facility^a^		
Daycare	30 (48.4)	
Residential	47 (75.8)	
Educational	4 (6.5)	
Other	9 (14.5)	
Size of facility (in number of people with profound intellectual and multiple disabilities)		
< 10	13 (21.0)	
10–49	31 (50.0)	
50–99	2 (3.2)	
≥ 100	16 (25.8)	
Use of other interventions in addition to Programma Perspectief^a^		
Yes	30 (54.5)	
No	25 (45.5)	

*Note:* Due to rounding, percentages may add up to more than 100%.

^a^ Results apply to all respondents except managers and team leads.

^b^ Multiple response options.

Almost half (44.8%) of the respondents indicated that the intervention had been implemented in their organisations two or more years ago. For 27.6%, the implementation had taken place less than 2 years ago, and for another 27.6%, the time since implementation is unknown. The majority (81.0%) of the respondents had attended a training session when they started working with the intervention. For about half of these respondents, the training had been provided by a trainer from an external organisation (53.2%). Most (58.6%) respondents who had taken the initial training had not attended any further training (58.6%). More than one third (37.1%) of these respondents nevertheless indicated a need for further training. Analysis of the open‐ended answers revealed that respondents experienced this need mainly because the initial knowledge had subsided, but also because they wanted to be able to evaluate the process of working with the intervention and discuss problems that had been encountered in practise. Respondents who indicated no need for further training (62.9%) explained that they were “still doing okay with it”, had recently attended the training, or did not consider it necessary to attend further training in light of their own profession or role.

### 3.1 Application of the Core Elements

Table 2 shows the extent to which respondents applied the core elements of the intervention.

**TABLE 2:** Application of the core elements of the intervention (%).

Normative statement^a^	*n*	Never *n* (%)	Sometimes *n* (%)	Usually *n* (%)	Always *n* (%)
I work based on the idea that people with profound intellectual and multiple disabilities are able to express their needs and desires in their relationships with others.	51	0 (0.0)	5 (9.8)	13 (19.4)	33 (64.7)
I work development‐oriented	51	0 (0.0)	4 (7.8)	22 (43.1)	25 (49.0)

Methodical approach	*n*	Disagree *n* (%)	Agree *n* (%)
In our organisation…			
…we work with personal profiles.	61	2 (3.3)	59 (96.7)
…we work with long‐term goals of 1–2 years.	62	2 (3.2)	60 (96.8)
…we work with long‐term goals of 6–12 months.	62	1 (1.6)	61 (98.4)
…we work with short‐term goals.	62	1 (1.6)	61 (98.4)
…we evaluate goals.	61	3 (4.9)	58 (95.1)

	*n*	Never *n* (%)	Sometimes *n* (%)	Usually *n* (%)	Always *n* (%)
In general, I work with goals.	51	1 (2.0)	3 (5.9)	21 (41.2)	26 (51.0)
Short‐term goals					
I set short‐term goals.^b^	31	7 (22.6)	7 (22.6)	7 (22.6)	10 (32.3)
In my experience, direct‐support professionals and allied healthcare professionals are capable of independently formulating correct short‐term goals.^c^	26	0 (0.0)	12 (46.2)	14 (53.8)	0
I help direct‐support professionals and allied healthcare professionals with formulating short‐term goals.^d^	20	0 (0.0)	7 (35.0)	12 (60.0)	1 (5.0)
The short‐term goals I set contribute to the long‐term goals.	44	1 (2.3)	1 (2.3)	14 (31.8)	28 (63.6)
Before setting short‐term goals, I set the Goal Attainment Scale (GAS).	44	14 (31.8)	8 (18.2)	9 (20.5)	13 (29.5)
I evaluate short‐term goals.	44	1 (2.3)	6 (13.6)	11 (25.0)	26 (59.1)

Interdisciplinary collaboration	*n*	Never *n* (%)	Sometimes *n* (%)	Usually *n* (%)	Always *n* (%)
I collaborate with colleagues within my own discipline.	51	0 (0.0)	6 (11.8)	17 (33.3)	28 (54.9)
I collaborate with colleagues outside of my own discipline.	51	3 (5.9)	8 (15.7)	20 (39.2)	20 (39.2)
I collaborate with relatives of people with profound intellectual and multiple disabilities.	51	4 (7.8)	15 (29.4)	14 (27.5)	18 (35.3)
I have consultations with colleagues from other disciplines and with relatives about formulating and evaluating the long‐term (1–2 years) goals of people with profound intellectual and multiple disabilities.	53	8 (15.1)	7 (13.2)	9 (17.0)	29 (54.7)
I have consultations with colleagues from other disciplines and with relatives about formulating and evaluating the long‐term (6–12 months) goals of people with profound intellectual and multiple disabilities.	54	9 (16.7)	8 (14.8)	11 (20.4)	26 (48.1)

*Note:* A higher percentage of “always” or “agree” represents a higher degree of applying the corresponding core element.

^a^ Results apply to all professionals except managers, team leads, physicians, and nurses.

^b^ Results apply to direct‐support professionals and allied healthcare professionals.

^c^ Results apply to health care psychologists, managers, and team leads.

^d^ Results apply to health care psychologists.

#### 3.1.1 Normative Statement and Methodical Approach

The majority of respondents indicated that they always or usually work according to the normative statement underlying the intervention. Moreover, almost all respondents indicated that in their organisation, the methodical approach of personal profiles, long‐term goals, short‐term goals, and evaluation of goals is applied.

Almost half (45.2%) of the direct‐support professionals and allied healthcare professionals stated that they never or sometimes set any short‐term goals. All healthcare psychologists, managers, and team leads indicated that direct‐support professionals and allied healthcare professionals are sometimes or usually capable of setting short‐term goals without help. Most often, help is provided by the healthcare psychologist. Respondents who did set short‐term goals indicated that these goals usually contribute to the long‐term goals. About half of the respondents said that they never or sometimes use the Goal Attainment Scale method when setting short‐term goals.

About half of the respondents (46.5%) reported that they evaluate short‐term goals after the prescribed period of 4–6 weeks. For other respondents, the period was longer (30.2%) or flexible (20.9%). The period after which a long‐term goal of 1–2 years is evaluated is mostly as prescribed (82.2%). For long‐term goals of 6–12 months, the period was as prescribed in slightly more than half (53.3%) of all cases.

#### 3.1.2 Interdisciplinary Collaboration

As shown in Table 2, respondents generally collaborate with colleagues within their own disciplines and, to a lesser extent, with colleagues outside of their own disciplines, including relatives. About one third of the respondents never or sometimes consulted with colleagues from other disciplines and with relatives about formulating and evaluating the long‐term goals of people with profound intellectual and multiple disabilities.

### 3.2 The Relationship Between the Core Elements and the Number of Perceived Effects for People With Profound Intellectual and Multiple Disabilities

The results reveal positive significant correlations between the degree of applying several core elements of the intervention and the total number of perceived effects for people with profound intellectual and multiple disabilities. A higher total number of perceived effects is related to more often working in a development‐oriented way (*ρ* = 0.344; *p* = 0.013), more often working with goals in general (*ρ* = 0.291; *p* = 0.038), more often setting (*ρ* = 0.370; *p* = 0.041) and evaluating short‐term goals (*ρ* = 0.421; *p* = 0.004), and less often providing assistance in setting short‐term goals by behavioural scientists (*ρ* = 0.526; *p* = 0.017). Overall, according to the results of a Mann–Whitney U test, more effects were perceived by respondents who evaluate short‐term goals within the prescribed 4–6 weeks (Mean Rank = 28.00), as compared to those who deviate from this theoretically prescribed period (Mean Rank = 16.78, U = 110.000; *p* = 0.003). See Supporting Information 3 for all significant and non‐significant results.

### 3.3 The Relationship Between Time Since Implementation and the Core Elements, Implementation Factors, and Perceived Effects

#### 3.3.1 Core Elements

With regard to the application of the core elements, for all but one of the items, Chi‐square tests revealed no significant differences between respondents working in organisations in which the intervention had been implemented more recently (less than 2 years ago) and those in which implementation had taken place less recently (two or more years ago). One Chi‐square test indicated that respondents working in organisations that had implemented the intervention less recently were more often present at interdisciplinary consultations on formulating and evaluating long‐term goals of 1–2 years (*χ* ^2^ = 13.788; *p* = 0.003). See Supporting Information 3 for all significant and non‐significant results.

#### 3.3.2 Barriers to and Facilitators of Implementation

**FIGURE 1:**
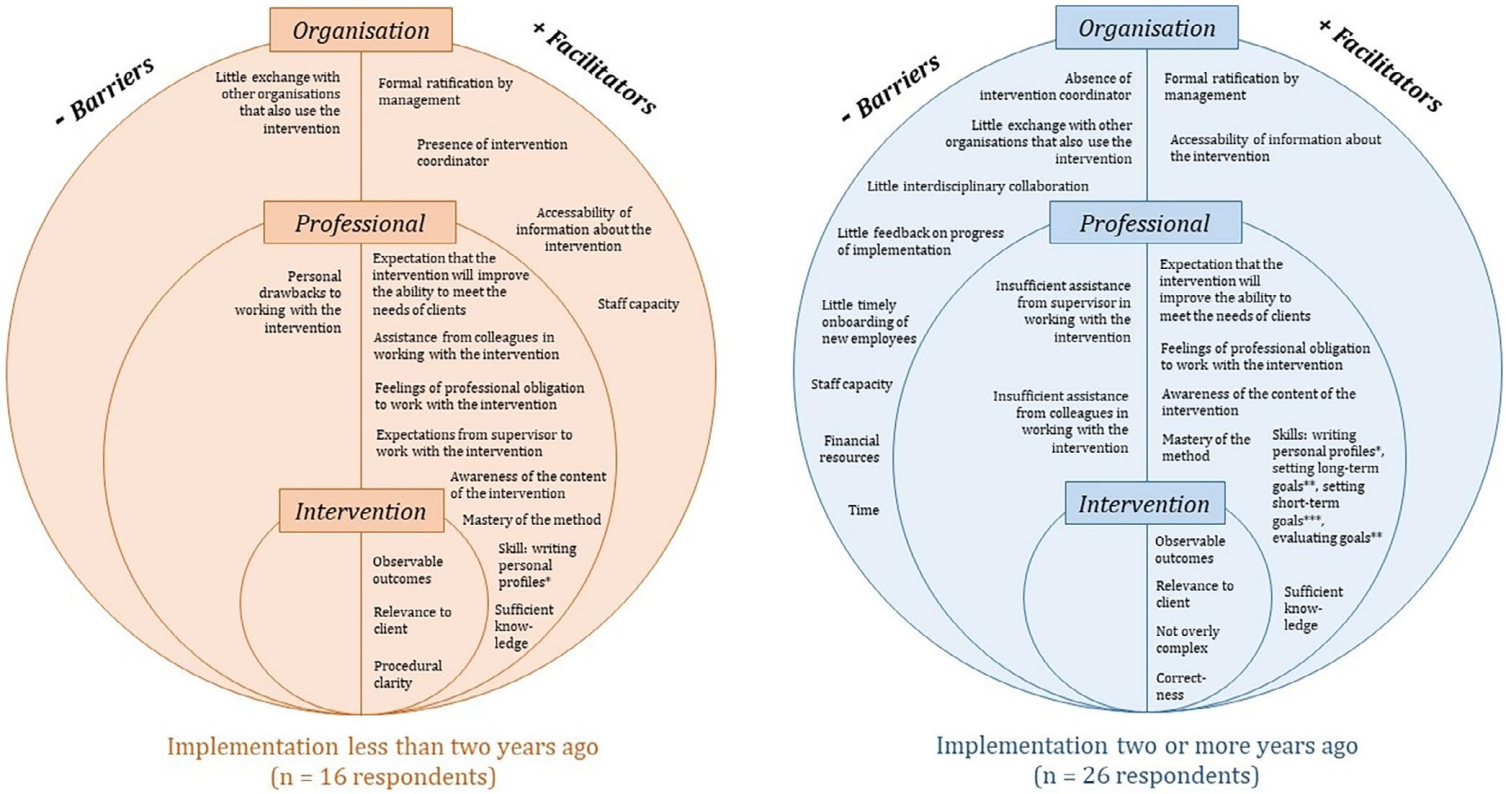
Implementation factors at the level of the intervention, professional and organisation when Programma Perspectief was implemented less than 2 years ago (left) and two or more years ago (right). Note: All items have response options 1–5, unless otherwise indicated. Items apply to all professionals except physicians and nurses, unless otherwise indicated. A factor is identified as a barrier if ≥ 20% disagreed or completely disagreed with the item in question. A factor is identified as a facilitator if ≥ 80% agreed or completely agreed. *Result applies only to health care psychologists. **Result does not apply to managers or team leads. ***Result applies only to direct‐support professionals and allied healthcare professionals.

The barriers to and facilitators of implementation of the intervention, as identified in this study, are presented in Figure 1. A distinction is made between respondents working in organisations that had implemented the intervention more recently (less than 2 years ago) and those in which it had been implemented less recently (two or more years ago). For a complete overview of the descriptive results for all factors, see Supporting Information 2.

Two barriers to implementation were identified amongst respondents working in organisations in which the implementation had taken place more recently: one at the level of the professional, with regard to perceived personal drawbacks due to working with the intervention (31.3%), and one at the level of the organisation, concerning the lack of exchange with other organisations (40.0%). Facilitators were identified at both the level of the intervention (e.g., observable outcomes [87.5%]), the professional (e.g., the expectation to better meet the needs of the person with disabilities [93.8%]), and the organisation (e.g., formal ratification by management about working with the intervention [88.9%]). In organisations that had implemented the intervention less recently, more barriers were identified, mostly at the organisational level (e.g., lack of time and staff [42.3%], lack of interdisciplinary collaboration [30.8%], and lack of feedback on the progress of the implementation [38.5%]). Facilitators emerged at the level of the intervention (e.g., the intervention is not perceived as overly complex [80.8%]) and the professional (e.g., several skills related to working with the intervention).

Several additional barriers and facilitators emerged from open‐ended responses. Regardless of time since implementation, one barrier that was mentioned was a lack of sufficient time for working with the intervention (*n* = 2 when implementation had taken place less than 2 years ago; *n* = 5 when implementation had taken place two or more years ago). Another barrier identified in organisations where implementation had taken place more recently was related to short‐term goals: too many short‐term goals and difficulty remembering and continuing to apply all of them (*n* = 8). Additional barriers mentioned in organisations where implementation had taken place less recently included staff turnover (*n* = 5), insufficient training (including for managers and health care psychologists [*n* = 4]), lack of involvement or motivation within the team (*n* = 3), lack of guidance on working with the intervention (*n* = 2), lengthy assessment procedures in the intervention (*n* = 2), and the application of the intervention with older people (*n* = 2). The facilitators that were mentioned differed with regard to time since implementation. In organisations where implementation had taken place more recently, respondents mentioned interdisciplinary (and other) collaboration and consultation (*n* = 4). Facilitators identified in organisations where the intervention had been implemented less recently included support for the intervention within the team and a shared vision (*n* = 7), initial/further training (*n* = 4), visibility of success (*n* = 2), clarity of the intervention (*n* = 2), and sufficient time (*n* = 2).

The results of a Mann–Whitney *U* test indicate that the total number of perceived barriers was significantly higher amongst respondents working in organisations where implementation had taken place less recently (mean rank = 24.79), as compared to those in which the intervention had been implemented more recently (mean rank = 16.16; U = 293.500; *p* = 0.025). No significant difference was found between the two groups in terms of the total number of perceived facilitators. See Supporting Information 3 for all significant and non‐significant results.

#### 3.3.3 Perceived Effects

The total number of perceived effects at both the level of the person with profound intellectual and multiple disabilities, the level of the professional, and the level of the organisation did not differ significantly according to time since implementation. See Supporting Information 3 for all the results.

## 4 Discussion

This study investigates the application of core elements of an intervention to support persons with profound intellectual and multiple disabilities in current practise and the association between application and the perceived effects. Further, it describes the association between the application, perceived barriers and facilitators, and the time since implementation. The results indicate that, regardless of time since implementation, professionals in practise applied some of the core elements of the intervention, especially the normative statement and the methodical approach of defining personal profiles and formulating and evaluating goals. The aspect of short‐term goals, which are to be formulated by direct‐support professionals and allied healthcare professionals, was implemented to a lesser extent. Results also showed that in many cases, no criteria were specified in advance for the evaluation of short‐term goals, and the period after which these goals are evaluated is longer than theoretically prescribed. The study further indicates that professionals generally collaborate with colleagues within their own disciplines, but to a lesser extent with colleagues from other disciplines and with relatives.

According to the results, when professionals (still) apply core elements to a greater extent, they perceive that the intervention has more effects for people with profound intellectual and multiple disabilities. No relationship was found between time since implementation and the perceived effects for the person with profound intellectual and multiple disabilities, the professional, or the organisation. However, a relationship was found between time since implementation and factors influencing implementation. Different types of barriers and facilitators were perceived as time passes. In addition, more barriers were perceived in organisations where implementation had taken place less recently, especially at the organisational level.

### 4.1 Theoretical Reflections

The observed strong fidelity to the normative statement, which consists of the development‐oriented and relationship‐oriented approaches, is in line with recent developments in the field of providing support to people with intellectual and developmental disabilities. The current Quality of Life Supports Paradigm acknowledges the importance and potential of development and personal autonomy with regard to the quality of life of people with intellectual and developmental disabilities, as well as recognises that these people need support in order to achieve this (Gómez Sánchez et al. 2021; Mumbardó‐Adam et al. 2023; Schalock et al. 2021). Programma Perspectief contributes to this paradigm, with good implementation being a prerequisite.

Although certain core elements of the intervention are being implemented well in current practise, the results of this study reveal that still improvements are needed in crucial key components such as working with short‐term goals and interdisciplinary collaboration. Previous studies already have indicated that some professionals continue to have difficulty setting good short‐term goals (Tadema et al. 2008), and the number of short‐term goals set is significantly lower than theoretically prescribed by the intervention (Vlaskamp and van der Putten 2009; Zijlstra 2003). Moreover, as reported by De Jong et al. (2022), setting goals is one of several challenges that care professionals experience in providing support to people with profound intellectual and multiple disabilities, as is interdisciplinary collaboration. Goal‐setting and interdisciplinary collaboration are essential in order to concretise the normative statement of development, relationship‐building, and personal autonomy for people with profound intellectual and multiple disabilities (Van der Putten et al. 2009; Vlaskamp et al. 2005). Furthermore, as confirmed in the current study, the application of the core elements is important to achieve the intended effects for these people (Poppes and Vlaskamp 2001; Vlaskamp and van der Putten 2009; Zijlstra 2003). It is therefore important to continue to devote special attention in practise to the aspects of goal‐setting and interdisciplinary collaboration.

In accordance with general literature on implementation (e.g., Bisschops et al. 2023; Damschroder et al. 2009; Grol et al. 2013; Kersten 2024) and previous research on intervention (Tadema et al. 2008; Zijlstra 2003), the results of this study suggest that barriers to and facilitators of implementation exist at multiple levels. Although employees seem to feel more competent in working with the intervention over time, more barriers emerge over time as well, particularly at the organisational level. Examples of such barriers include a lack of interdisciplinary collaboration, a lack of feedback on the progress of the implementation, a failure to onboard new staff in a timely manner, and a lack of resources in terms of time, money, and personnel. Another barrier has to do with the inability to obtain enough assistance from the supervisor when working with the intervention.

The results of this study indicate that the long‐term implementation of this evidence‐based support programme requires a specific focus on organisational‐level factors. The existing literature suggests that reducing organisational barriers can benefit the core element of interdisciplinary (or other) collaboration (Rawlinson et al. 2021). The focus on organisational factors implies an important role for managers in terms of providing preconditions for working with the intervention and providing support. This observation is in line with the findings of a previous study by Luijkx and Metsemakers (2008), who identify support from management as an important factor in the sustainable implementation of the intervention. It is also consistent with implementation studies identifying organisational factors (e.g., support and commitment from (higher) management, continued monitoring and evaluation of the implementation, the presence of sufficient staff and financial resources, and facilitation of training) as being important to sustaining implementation (Cowie et al. 2020; Damschroder et al. 2009; Gruen et al. 2008; Scheirer 2005; Wensing et al. 2020). In light of recent developments in the Netherlands regarding labour shortages in the support for people with intellectual disabilities (Peters et al. 2024), support for this already vulnerable group is likely to come under even more pressure, making it especially important to invest in attracting and retaining good personnel, as well as in training.

### 4.2 Methodological Reflections

A strength of this study is that it utilises knowledge and experience from the field to the fullest extent. Professionals were involved in designing the questionnaire and recruiting respondents on a broad scale. As a result of this involvement, it was possible to adapt the study well to the target group and the broader field.

Despite the fact that the questionnaire was anonymous, respondents may have given socially desirable answers that did not fully represent real practise. A follow‐up study could substantiate the results of this survey by analysing the content of the Individual Educational Plans developed within the intervention (e.g., Vlaskamp and van der Putten 2009) and/or observations in practise to track the behaviour of health care professionals and to analyse to what extent this is in line with the theoretically described components (e.g., studies on Active support, Aspling et al. 2024).

Several comments can be made with regard to the interpretation of the results of this study. First, the research was conducted in healthcare organisations within the network of the Academic Collaborative Centre for People with Profound Intellectual and Multiple Disabilities. These organisations may be more positive about and concerned with quality management and evidence‐based work compared to organisations that are not involved in such a network. Therefore, the results may be biassed in a positive direction. Second, the participating organisations are relatively large‐scale residential care facilities. It is therefore appropriate to question the extent to which the findings of this study might also apply to small‐scale institutions or educational institutions. Because insight into the population size of professionals working with the intervention is missing, we were not able to estimate the power of this study. The number of respondents (*n* = 62) who completed the questionnaire is generally seen as rather low. However, research on people with PIMD is more often faced with small sample sizes, explained by a limited number of people with PIMD supported by the intervention, and the professionals who support them do not always have time to participate in research studies (Maes et al. 2021).

It is also important to note that the perspectives of relatives were not included in the study, despite their important role in supporting people with profound intellectual and multiple disabilities (Jansen et al. 2013; Kruithof et al. 2024; Van Beurden et al. 2024). National policies are seeking to improve the matching of support for people with disabilities to their own needs, as well as those of their relatives (Government of the Netherlands 2022). Although a questionnaire was initially developed for relatives, the response was insufficient. This may be caused by relatives not seeing it as a priority to contribute to research. However, it may also be a sign that relatives were not sufficiently engaged in working with the intervention, and did not feel addressed by the call for the questionnaire, for example, because they are not familiar with which interventions the healthcare organisation uses. In future research, it will be important to include the perspectives of relatives.

### 4.3 Recommendations

In general, after initial implementation and in the long term, professionals do apply the core elements of the intervention Programma Perspectief—a promising intervention, the effects of which continue to be perceived by professionals. In line with the literature on implementation, however, the results of this study also indicate that a focus on implementing and sustaining the intervention remains necessary over time (Birken et al. 2020; Hailemariam et al. 2019; Shelton et al. 2018). The findings suggest that the implementation of an intervention such as Programma Perspectief is an ongoing process, and one that does not end after 2 years. Areas of focus include working with short‐term goals and interdisciplinary collaboration, as well as the increasing emergence of barriers to implementation over time. Given that such barriers are especially likely to exist at the organisational level, management has an important role to play in providing guidance, offering support, and creating the preconditions for working with the intervention. However, as the support for people with profound intellectual and multiple disabilities is characterised by interdisciplinarity, integrated care and 24/7 care, optimal implementation of the intervention requires commitment and adjustment from a variety of professionals, disciplines, and layers within the organisation (Bisschops et al. 2022; Kersten et al. 2018).

Implementation strategies can help overcome barriers and improve the implementation and sustainment of an intervention (Proctor et al. 2013). In the literature, a wide variety of implementation strategies exists, with strategies being categorised in different ways (Wensing et al. 2020). Examples include educational strategies (e.g., conducting ongoing training) and evaluative strategies (e.g., providing feedback or quality monitoring) (Waltz et al. 2015). However, in the support for people with intellectual disabilities, there is a lack of knowledge when it comes to the successful implementation of interventions (Bisschops 2024); scientific knowledge on implementation in curative care cannot be generalised to long‐term care (Bisschops et al. 2023; Kersten et al. 2018). Although this study was conducted within a specific target group and intervention, it also provides general knowledge on implementation and intervention fidelity supporting individuals with specialised support needs. Therefore, this research can contribute to the theoretical basis of implementation science, especially in long‐term care and support (Bisschops et al. 2022). In order for people with profound intellectual and multiple disabilities to receive optimal support, further research should focus on identifying which (combination of) implementation strategies are suitable to improve the implementation of the intervention, tailoring these strategies to the areas of focus found in the current study, and monitoring the effectiveness over time. These future studies, however, must be executed from a dynamical point of view in which we must balance between intervention fidelity and adaptations. Because too much emphasis on implementing the intervention correctly (fidelity) would hamper sustainability and ongoing improvement, customisation and optimisation of interventions are necessary (Chambers et al. 2013; Chambers and Norton 2016). Any such future research should be conducted in collaboration with stakeholders in the field, including relatives.

## Ethics Statement

The Ethics Committee of Behavioural and Social Sciences of University of Groningen granted permission to conduct the study (8 Oct. 2023, code: UG‐2223‐GMW‐S‐000007).

## Conflicts of Interest

The authors declare no conflicts of interest.

## Supporting information


**Data S1.**Supporting Information.

## Data Availability

The data that support the findings of this study are available from the corresponding author upon reasonable request.

## Endnotes

^1^
A health care psychologist is a support professional with a master degree in the behavioural and social sciences (e.g., psychology or special needs education in Dutch ‘Orthopedagogiek’). This professional is one of the leading disciplines related to the quality of support and individualised support planning including assessment and interventions.

^2^
This means that people with profound intellectual and multiple disabilities have intellectual disabilities that are so severe that none of the existing standardised tests can give a valid estimate of the degree of intellectual disability. In addition, they have a severe or profound motor disability, and their communication is often non‐verbal. Given the severity and type of impairments, these people are dependent on support from others in all aspects of daily life.
