# Supplementary material for: An Ongoing Process: The Implementation of an Intervention for People With Profound Intellectual and Multiple Disabilities Over Time
Source: J Appl Res Intellect Disabil. 2025 Jul 16;38(4):e70098. doi: 10.1111/jar.70098 (PMC12265036; doi:10.1111/jar.70098)
Supplement: Supplementary file 1 — Data S1.Supporting Information. [file JAR-38-e70098-s001.docx]

**Supplementary Material 1. Computation of ratio variables for effects at various levels**

Table 1. Computation of the total number of perceived effects at the level of the person with profound intellectual and multiple disabilities, the professional, and the organisation.

| Variable | Items | Computation |
| --- | --- | --- |
| Total number of perceived effects at the level of the person with profound intellectual and multiple disabilities | My clients have progressed overall.  My clients are better understood.  Communication with my clients has improved.  My clients’ mood has improved.  My clients are more environment-oriented.  My clients are more active.  My clients are better able to connect with others.  My clients’ physical posture has improved.  My clients are more present (more alert).  My clients’ health has improved. | For each item: 2 points for the answer ‘yes, majority’, 1 point for the answer ‘yes, minority’ and 0 points for the answers ‘no’ or ‘no opinion/don’t know’ |
| Total number of perceived effects at the level of the professional | I work more based on the idea that people with profound and intellectual disabilities are able to express their needs and desires in their relationships with others.  I work more methodically and systematically, with goals.  I work more collaboratively with other professionals.  I align my goals more with jointly established long term goals.  I am content with my work.  I get more feelings of fulfillment out of my work.  I am able to give more substance to the support of clients. | For each item: 1 point for the answers ‘agree’ and ‘completely agree’, 0 points for the answers ‘disagree’, ‘completely disagree’ and ‘no opinion’ |
| Total number of perceived effects at the level of the organisation | Programma Perspectief contributes to collaboration with other professionals within my discipline.  Programma Perspectief contributes to collaboration with other professionals outside of my discipline.  Programma Perspectief contributes to collaboration with relatives of clients.  Programma Perspectief contributes to more professional work.  Programma Perspectief contributes to more consistency in the support of clients.  Programma Perspectief helps to make better choices in the support of clients. | For each item: 1 point for the answers ‘agree’ and ‘completely agree’, 0 points for the answers ‘disagree’, ‘completely disagree’ and ‘no opinion’ |

**Supplementary Material 2. Implementation factors by time since implementation**

1. **Group: implementation less than two years ago**

Table 2. Implementation factors in organisations that implemented Programma Perspectief (PP) less than two years ago. Percentages of barriers and facilitators in **bold.**

| Factor | Item | N | % (completely) disagree | %  neutral | % (completely) agree |
| --- | --- | --- | --- | --- | --- |
| Intervention level  Procedural clarity**  Correctness  Completeness  Complexity  Compatibility  Observability  Relevance to client | PP clearly describes the activities I should perform and in which order.  PP is based on factually correct knowledge.  PP provides all the information and materials needed to work with it properly.  PP is too complex for me to use.  PP is a good match for how I am used to working.  The outcomes of using PP are clearly observable.  I think PP is relevant for my clients. | 13  16  16  16  16  16  16 | 0.0  0.0  6.3  12.5  12.5  6.3  6.3 | 15.4  25.0  25.0  12.5  25.0  6.3  13.8 | **84.6**  75.0  68.8  75.0  62.5  **87.5**  **93.8** |
| Professional level  Personal benefits  Personal drawbacks  Outcome expectations  Professional obligation  Social support: co-workers  Social support: supervisor  Subjective norm: supervisor  Self-efficacy: mastery of the working method  Self-efficacy: long-term goal 1-2 years**  Self-efficacy: long-term goal 6-12 months**  Self-efficacy: short-term goal ***  Self-efficacy: GAS***  Self-efficacy: evaluating and adjusting goals**  Self-efficacy: writing a personal profile****  Knowledge  Content awareness (1-4)  Application older clients *  Application clients with more additional impairments * | Using PP has personal benefits for me.  Using PP has personal drawbacks for me.  I expect that PP helps us to better meet the needs of clients so that they can develop to their full potential.  As a professional, I feel that it is my responsibility to use PP.  I can count on adequate assistance from my colleagues if I need it to use PP.  I can count on adequate assistance from my supervisor if I need it to use PP.  My supervisor expects me to use PP.  I master the working method of PP.  I am good at setting a long-term goal of 1-2 years for a client.  I am good at setting a long-term goal of 6-12 months for a client.  I am good at setting a short term goal for a client.  I am good at Goal Attainment Schaling.  I am good at evaluating goals and adjusting them if necessary.  I am good at writing a personal profile.  I have sufficient knowledge to use PP.  To wat extent are you aware of the content of PP?  It is difficult to use PP as intended when it comes to older clients.  It is difficult to use PP as intended when it comes to clients with more additional impairments. | 16  16  16  16  16  16  16  16  13  13  12  12  13  1  16  16  16  16 | 0.0  **31.3**  0.0  0.0  6.3  0.0  0.0  0.0  15.4  15.4  16.7  16.7  15.4  0.0  0.0  6.3  6.3  18.8 | 56.3  56,3  6.3  18.8  0.0  18.8  18.8  12.5  38.5  38.5  33.3  50.0  30.8  0.0  18.8  -  25.0  25.0 | 43.8  12,5  **93.8**  **81.3**  **93.8**  **81.3**  **81.3**  **87.5**  46.2  46.2  50.0  33.3  53.8  **100.0**  **81.3**  **93.8**  68.8  56.3 |
| Organisational level  Formal ratification by management (1-2)  Timely onboarding of new employees  Staff capacity  Financial resources  Time available  Material resources and facilities  Coordinator (1-2)  Accessibility of information (1-2)  Performance feedback  Information exchange with other organisations (1-2)  Interdisciplinary collaboration* | In my organisation, the management has set up formal arrangements relating to the use of PP (in policy plans, work plans and so on).  In my organisation, new employees are introduced to PP in a timely manner.  In my organisation, there are enough people to use PP as intended.  In my organisation, there are enough financial resources available to use PP as intended.  My organisation provides me with enough time to include PP as intended in my day-to-day work.  My organisation provides me with enough materials and other resources or facilities necessary for the use of PP as intended.  In my organisation, one or more people have been designated to coordinate the process of implementing PP.  It is easy for me to find information in my organisation about using PP as intended.  In my organisation, feedback on the progress of implementing PP is regularly provided.  In my organisation, we talk about PP with other organisations that also use PP.  In my organisation, there is enough interdisciplinary collaboration to use PP as intended. | 9  16  16  16  16  16  11  13  16  5  16 | 11.1  0.0  12.5  0.0  12.5  6.3  0.0  0.0  6.3  **40.0**  6.3 | -  31.3  6.3  50.0  37.5  18.8  -  -  31.3  -  31.3 | **88.9**  68.8  **81.3**  50.0  50.0  75.0  **100.0**  **100.0**  62.5  60.0  62.5 |

*Note:* All items have response options 1–5 unless otherwise indicated. Items apply to all professionals except physicians and nurses unless otherwise indicated. A factor is identified as a barrier if ≥ 20% disagreed or completely disagreed with the item in question. A factor is identified as a facilitator if ≥ 80% agreed or completely agreed.

*** Addition to MIDI-items, based on previous research on Programma Perspectief*.*

**** Result does not apply to managers or team leads.

*** Result only applies to direct support professionals and therapists.

**** Result only applies to behavioural scientists.

1. **Group: implementation two or more years ago**

Table 3. Implementation factors in organisations that implemented Programma Perspectief (PP) two or more years ago. Percentages of barriers and facilitators in **bold.**

| Factor | Item | N | % (completely) disagree | %  neutral | % (completely) agree |
| --- | --- | --- | --- | --- | --- |
| Intervention level  Procedural clarity**  Correctness  Completeness  Complexity  Compatibility  Observability  Relevance to client | PP clearly describes the activities I should perform and in which order.  PP is based on factually correct knowledge.  PP provides all the information and materials needed to work with it properly.  PP is too complex for me to use.  PP is a good match for how I am used to working.  The outcomes of using PP are clearly observable.  I think PP is relevant for my clients. | 24  26  26  26  26  26  26 | 16.7  3.8  15.4  15.4  15.4  7.7  3.8 | 25.0  15.4  23.1  3.8  11.5  7.7  15.4 | 58.3  **80.8**  61.5  **80.8**  73.1  **84.6**  **80.8** |
| Professional level  Personal benefits  Personal drawbacks  Outcome expectations  Professional obligation  Social support: co-workers  Social support: supervisor  Subjective norm: supervisor  Self-efficacy: mastery of the working method  Self-efficacy: long-term goal 1-2 years**  Self-efficacy: long-term goal 6-12 months**  Self-efficacy: short-term goal ***  Self-efficacy: GAS***  Self-efficacy: evaluating and adjusting goals**  Self-efficacy: writing a profile****  Knowledge  Content awareness (1-4)  Application older clients *  Application clients with more additional impairments * | Using PP has personal benefits for me.  Using PP has personal drawbacks for me.  I expect that PP helps us to better meet the needs of clients so that they can develop to their full potential.  I feel that it is my responsibility as a professional to use PP.  I can count on adequate assistance from my colleagues if I need it to use PP.  I can count on adequate assistance from my supervisor if I need it to use PP.  My supervisor expects me to use PP.  I master the working method of PP.  I am good at setting a long-term goal of 1-2 years for a client.  I am good at setting a long-term goal of 6-12 months for a client.  I am good at setting a short term goal for a client.  I am good at Goal Attainment Schaling.  I am good at evaluating goals and adjusting them if necessary.  I am good at writing a personal profile.  I have sufficient knowledge to use PP.  To wat extent are you aware of the content of PP?  It is difficult to use PP as intended when it comes to older clients.  It is difficult to use PP as intended when it comes to clients with more additional impairments. | 26  26  26  26  26  26  26  26  24  24  12  12  24  12  26  26  26  16 | 11.5  11.5  0.0  3.8  **23.1**  **34.6**  11.5  3.8  0.0  0.0  8.3  16.7  4.2  0.0  3.8  3.8  15.4  7.7 | 26.9  30.8  7.7  11.5  7.7  38.5  26.9  3.8  4.2  4.2  8.3  33.3  8.3  0.0  15.4  -  23.1  15.4 | 61.5  57.7  **92.3**  **84.6**  69.7  26.9  61.5  **92.3**  **95.8**  **95.8**  **83.3**  50.0  **87.5**  **100.0**  **80.8**  **96.2**  61.5  76.9 |
| Organisational level  Formal ratification by management (1-2)  Timely onboarding of new employees  Staff capacity  Financial resources  Time available  Material resources and facilities  Coordinator (1-2)  Accessibility of information (1-2)  Performance feedback  Information exchange with other organisations (1-2)  Interdisciplinary collaboration * | In my organisation, the management has set up formal arrangements relating to the use of PP (in policy plans, work plans and so on).  In my organisation, new employees are introduced to PP in a timely manner.  In my organisation, there are enough people to use PP as intended.  In my organisation, there are enough financial resources available to use PP as intended.  My organisation provides me with enough time to include PP as intended in my day-to-day work.  My organisation provides me with enough materials and other resources or facilities necessary for the use of PP as intended.  In my organisation, one or more people have been designated to coordinate the process of implementing PP.  It is easy for me to find information in my organisation about using PP as intended.  In my organisation, feedback on the progress of implementing PP is regularly provided.  In my organisation, we talk about PP with other organisations that also use PP.  In my organisation, there is enough interdisciplinary collaboration to use PP as intended. | 16  26  26  26  26  26  22  25  26  15  26 | 0.0  **42.3**  **38.5**  **34.6**  **42.3**  19.2  **22.7**  4.0  **38.5**  **80.0**  **30.8** | -  26.9  15.4  26.9  23.1  15.4  -  -  19.2  -  11.5 | **100.0**  30.8  46.2  38.5  34.6  65.4  77.3  **96.0**  42.3  20.0  5.7 |

*Note:* All items have response options 1–5 unless otherwise indicated. Items apply to all professionals except physicians and nurses unless otherwise indicated. A factor is identified as a barrier if ≥ 20% disagreed or completely disagreed with the item in question. A factor is identified as a facilitator if ≥ 80% agreed or completely agreed.

*** Addition to MIDI-items, based on previous research on Programma Perspectief*.*

**** Result does not apply to managers or team leads.

*** Result only applies to direct support professionals and therapists.

**** Result only applies to behavioural scientists.

**Supplementary Material 3. Bivariate analyses**

1. **The relationship between the core elements and the number of perceived effects for people with profound intellectual and multiple disabilities**

Table 4. Spearman correlations between various core elements and the total number of perceived effects at the level of the person with profound intellectual and multiple disabilities. Significant results with p-values in **bold**.

| Variable 1 | Variable 2 | Spearman’s  rho | p |
| --- | --- | --- | --- |
| Total number of perceived effects at the level of the person with profound intellectual and multiple disabilities | I work based on the idea that people with profound and intellectual disabilities are able to express their needs and desires in their relationships with others.  I work development-oriented.  In general, I work with goals.  I collaborate with colleagues within my own discipline.  I collaborate with colleagues outside of my own discipline.  I collaborate with relatives of people with profound intellectual and multiple disabilities.  Do you set short-term goals for people with profound intellectual and multiple disabilities?  In your experience, are direct support professionals and therapists capable of independently formulating correct short-term goals?  Do you ever help direct support professionals and therapists with formulating short-term goals?  The short-term goals I set contribute to the long-term goals.  Before setting short-term goals, I set the Goal Attainment Scale (GAS).  I evaluate short-term goals.  I have consultations with colleagues from other disciplines and with relatives about formulating and evaluating the long-term (1–2 years) goals of people with profound intellectual and multiple disabilities.  I have consultations with colleagues from other disciplines and with relatives about formulating and evaluating the long-term (6-12 months) goals of people with profound intellectual and multiple disabilities. | 0.193  0.344  0.291  0.165  0.037  0.118  0.370  0.367  0.526  0.149  0.074  0.421  0.167  0.273 | 0.174  **0.013**  **0.038**  0.247  0.795  0.410  **0.041**  0.111  **0.017**  0.335  0.634  **0.004**  0.250    0.055 |

Table 5. Mann-Whitney U tests for differences in the total number of perceived effects at the level of the person with profound intellectual and multiple disabilities between respondents who do and do not evaluate goals within the specified period of time. Significant results with p-values in **bold**.

| Variabele | Group | Mean Rank | U | p |
| --- | --- | --- | --- | --- |
| Total number of perceived effects at the level of the person with profound intellectual and multiple disabilities | Duration of long-term goal of 1-2 years  1 or 2 years  Not in accordance to theory  Duration of long-term goal of 6-12 months  6 to 12 months  Not in accordance to theory  Duration of short-term goal  4 to 6 weeks  Not in accordance to theory | 19.57  22.90  21.06  18.30  28.00  16.78 | 99.500  154.500  110.000 | 0.554  0.466  **0.003** |

1. **The relationship between time since implementation and the core elements, implementation factors, and perceived effects at various levels**

Table 6. Chi-square tests for differences in the degree of working according to the core elements between respondents employed at organisations where PP was implemented less than two years ago and two or more years ago. Significant results with p-values in **bold**.

| Variabele | Answer | | Implementation  <2 years ago | Implementation  ≥2 years ago | Total | χ2 | df | p |
| --- | --- | --- | --- | --- | --- | --- | --- | --- |
| I work based on the idea that people with profound and intellectual disabilities are able to express their needs and desires in their relationships with others. | Sometimes  Usually  Always | Observed  Expected  Observed  Expected  Observed  Expected | 1  1,4  7  3,9  5  7,7 | 3  2,6  4  7,1  17  14,3 | 4  4  11  11  22  22 | 5.587 | 2 | 0.061 |
| I work development-oriented. | Sometimes  Usually  Always | Observed  Expected  Observed  Expected  Observed  Expected | 0  1,1  8  5,3  5  6,7 | 3  1,9  7  9,7  14  12,3 | 3  3  15  15  19  19 | 4.453 | 3 | 0.108 |
| In general, I work with goals. | Never  Sometimes  Usually  Always | Observed  Expected  Observed  Expected  Observed  Expected  Observed  Expected | 0  0,4  0  0,7  8  5,6  5  6,3 | 1  0,6  2  1,3  8  10,4  13  11,7 | 1  1  2  2  16  16  18  18 | 3.604 | 2 | 0.308 |
| I collaborate with colleagues within my own discipline. | Sometimes  Usually  Always | Observed  Expected  Observed  Expected  Observed  Expected | 0  1,4  5  4,6  8  7 | 4  2,6  8  8,4  12  13 | 4  4  13  13  20  20 | 2.437 | 2 | 0.296 |
| I collaborate with colleagues outside of my own discipline. | Never  Sometimes  Usually  Always | Observed  Expected  Observed  Expected  Observed  Expected  Observed  Expected | 2  1,1  4  2,1  5  5,3  2  4,6 | 1  1,9  2  3,9  10  9,7  11  8,4 | 3  3  6  6  15  15  13  13 | 6.173 | 3 | 0.104 |
| I collaborate with relatives of people with profound intellectual and multiple disabilities. | Never  Sometimes  Usually  Always | Observed  Expected  Observed  Expected  Observed  Expected  Observed  Expected | 2  1,1  2  2,8  6  4,2  3  4,9 | 1  1,9  6  5,2  6  7,8  11  9,1 | 3  3  8  8  12  12  14  14 | 3.987 | 3 | 0.263 |
| Do you set short-term goals for people with profound intellectual and multiple disabilities? | Never  Sometimes  Usually  Always | Observed  Expected  Observed  Expected  Observed  Expected  Observed  Expected | 5  3  2  2,5  1  2  4  4,5 | 1  3  3  2,5  3  2  5  4,5 | 6  6  5  5  4  4  9  9 | 3.978 | 3 | 0.264 |
| In your experience, are direct support professionals and therapists capable of independently formulating correct short-term goals? | Sometimes  Usually | Observed  Expected  Observed  Expected | 1  1,9  3  2,1 | 7  6,1  6  6,9 | 8  8  9  9 | 1.022 | 1 | 0.312 |
| Do you ever help direct support professionals and therapists with formulating short-term goals? | Sometimes  Usually  Always | Observed  Expected  Observed  Expected  Observed  Expected | 0  0,3  1  0,6  0  0,1 | 4  3,7  7  7,4  1  0,9 | 4  4  8  8  1  1 | 0.677 | 2 | 0.713 |
| The short-term goals I set contribute to the long-term goals. | Never  Sometimes  Usually  Always | Observed  Expected  Observed  Expected  Observed  Expected  Observed  Expected | 0  0,3  0  0,3  1  2,6  7  4,9 | 1  0,7  1  0,7  9  7,4  12  14,1 | 1  1  1  1  10  10  19  19 | 3.209 | 3 | 0.361 |
| Before setting short-term goals, I set the Goal Attainment Scale (GAS). | Never  Sometimes  Usually  Always | Observed  Expected  Observed  Expected  Observed  Expected  Observed  Expected | 1  2,3  1  1,3  2  1,3  4  3,1 | 8  6,7  4  3,7  3  3,7  8  8,9 | 9  9  5  5  5  5  12  12 | 1.984 | 3 | 0.576 |
| I evaluate short-term goals. | Never  Sometimes  Usually  Always | Observed  Expected  Observed  Expected  Observed  Expected  Observed  Expected | 1  0,3  1  1,0  0  1,5  6  5,2 | 0  0,7  3  3,0  6  4,5  14  14,8 | 1  1  4  4  6  6  20  20 | 5.147 | 3 | 0.161 |
| I have consultations with colleagues from other disciplines and with relatives about formulating and evaluating the long-term (1–2 years) goals of people with profound intellectual and multiple disabilities. | Never  Sometimes  Usually  Always | Observed  Expected  Observed  Expected  Observed  Expected  Observed  Expected | 6  2,2  1  1  0  1,9  6  7,8 | 0  3,8  2  1,9  5  3,1  15  13,2 | 6  6  3  3  5  5  21  21 | 13.788 | 3 | **0.003** |
| I have consultations with colleagues from other disciplines and with relatives about formulating and evaluating the long-term (6-12 months) goals of people with profound intellectual and multiple disabilities. | Never  Sometimes  Usually  Always | Observed  Expected  Observed  Expected  Observed  Expected  Observed  Expected | 6  2,5  1  1,8  1  1,8  5  6,9 | 1  1,5  4  3,2  4  3,2  14  12,1 | 7  7  5  5  5  5  19  19 | 9.381 | 3 | **0.025** |
| After what period of time is the long-term goal of 1-2 years usually evaluated? | After 1 or 2 years  Not in accordance to theory | Observed  Expected  Observed  Expected | 6  4,9  0  1,1 | 17  18,1  5  3,9 | 23  23  5  5 | 1.660 | 1 | 0.198 |
| After what period of time is the long-term goal of 6-12 months usually evaluated? | After 6 to 12 months  Not in accordance to theory | Observed  Expected  Observed  Expected | 5  4,0  1  2,0 | 13  14,0  8  7,0 | 18  18  9  9 | 0.964 | 1 | 0.326 |
| After what period of time are the short-term goals usually evaluated? | After 4 to 6 weeks  Not in accordance to theory | Observed  Expected  Observed  Expected | 5  3,3  2  3,7 | 9  10,7  14  12,3 | 14  14  16  16 | 2.249 | 1 | 0.134 |

Table 7. Mann-Whitney U tests for differences in the total number of perceived barriers and facilitators between respondents employed at organisations where PP was implemented less than two years ago and two or more years ago. Significant results with p-values in **bold**.

| Variable | Group | Mean Rank | U | p |
| --- | --- | --- | --- | --- |
| Total number of perceived barriers | Implementation <2 years ago  Implementation ≥2 years ago | 16,16  24,79 | 293.500 | **0.025** |
| Total number of perceived facilitators | Implementation <2 years ago  Implementation ≥2 years ago | 20,47  22,13 | 224.500 | 0.669 |

Table 8. Mann-Whitney U tests for differences in the total number of perceived effects at the level of the person with profound intellectual and multiple disabilities, the professional and the organisation between respondents employed at organisations where PP was implemented less than two years ago and two or more years ago. Significant results with p-values in **bold**.

| Variable | Group | Mean Rank | U | p |
| --- | --- | --- | --- | --- |
| Total number of perceived effects at the level of the person with profound intellectual and multiple disabilities | Implementation <2 years ago  Implementation ≥2 years ago | 18.42  19.31 | 163.500 | 0.814 |
| Total number of perceived effects at the level of the professional | Implementation <2 years ago  Implementation ≥2 years ago | 17.50  19.81 | 175.500 | 0.540 |
| Total number of perceived effects at the level of the organisation | Implementation <2 years ago  Implementation ≥2 years ago | 22.63  20.81 | 190.000 | 0.597 |
